# Music and Emotions in Non-Human Animals from Biological and Comparative Perspectives

**DOI:** 10.3390/ani14101491

**Published:** 2024-05-17

**Authors:** Juliana Zapata-Cardona, Maria Camila Ceballos, Berardo de Jesús Rodríguez

**Affiliations:** 1 Grupo de Investigación Patobiología QUIRON, Escuela de Medicina Veterinaria, Universidad de Antioquia, Calle 70 No. 52-21, Medellín 50010, Colombia; berardo.rodriguez@udea.edu.co; 2 Faculty of Veterinary Medicine, University of Calgary, Clinical Skills Building, 11877-85th Street NW, Calgary, AB T3R 1J3, Canada

**Keywords:** animal welfare, musical environmental enrichment, emotionality

## Abstract

**Simple Summary:**

For humans, music is a powerful tool of emotional communication, conveying affective states and modulating physiological states in ways that can influence well-being. Understanding that emotionality is not an exclusively human trait, as it is also recognized in non-human species, it is natural to assume that the intrinsic power of music to modulate the psychophysiological state may be trans-specific. In this way, music can be a powerful tool for enriching the environment and improving the welfare of captive animals, especially farm animals. As there is very limited information on non-human animals, the aim is to review what is known from a human comparative perspective, arguments that support its use, and the potential to use music in non-human species.

**Abstract:**

The effects of sound stimulation as a sensorial environmental enrichment for captive animals have been studied. When appropriately implemented for farm animals, it can improve welfare, health, and productivity. Furthermore, there are indications that music can induce positive emotions in non-human animals, similar to humans. Emotion is a functional state of the organism involving both physiological processes, mediated by neuroendocrine regulation, and changes in behavior, affecting various aspects, including contextual perception and welfare. As there is very limited information on non-human animals, the objective of this review is to highlight what is known about these processes from human biological and comparative perspectives and stimulate future research on using music to improve animal welfare.

## 1. Introduction

As there is limited information on non-human animals, this review presents a theoretical and contextual approach to music-induced emotions in non-human animals based on human biological and comparative perspectives. Until recently, emotions generated by music were not considered biological functions. Therefore, their effects on emotionality modulation in animals is a developing research field, with many knowledge gaps.

The study of emotion has always been controversial, particularly for non-human animals. Despite the reluctance to attribute emotions to non-human animals and to limit behavioral changes to functional interpretations, there are considerable data that suggest a need to rethink these considerations.

Darwin was the first to argue that emotions and related expressive behaviors are phylogenetically ancient and part of a set of mechanisms that humans share with other animals [1]. Many animal behavior scientists have advocated for the recognition of emotionality, facilitating acceptance of labeling certain animal actions as “emotional” [2,3,4,5,6]. In 1997, the Treaty of Amsterdam recognized the capacity of animals to be sentient. This amended previous European Union treaties and established that European Union public policies related to agriculture, transport, the domestic market, and research must consider animals’ needs and promote their welfare [7]. This, coupled with advances in neurophysiology and neuroimaging, facilitates the assessment of neurological activity and interactions during exposure to various stimuli and reveals a deep biological basis for emotionality [8,9,10,11].

Recognition of emotions in animals is of great importance for studying animal welfare. The Brambell Committee Report (1965) recognized the role of mental processes in animal welfare and stated that “welfare is a wide term that embraces both the physical and mental well-being of the animal. Any attempt to evaluate welfare, therefore, must take into account the scientific evidence available concerning the feelings of animals that can be derived from their structure and functions and also from their behaviour” [12]. “Animal welfare is defined as the physical and mental state of an animal in relation to the conditions in which it lives and dies” [13]. Focusing only on optimizing basic functioning to improve animal welfare is inadequate, as animal welfare includes affective states and natural behaviors [14]. Multiple non-human species are sentient and feel pain, distress, and pleasant emotions [15]. Such approaches fostered interest in emotions as a fundamental component of welfare, as evidenced by subsequent advances that led to the emergence of disciplines, such as affective neuroscience [4] and positive psychology, exploring emotions, their origin and meaning, and their role as primary components of subjective well-being [16].

Music can evoke various emotions in humans, with similar responses observed in other animals [17,18]. This has prompted explorations of the origins of music and causal relationships between acoustic characteristics of music and associated emotional responses [18]. Several theories and hypotheses have been proposed from functional, mechanistic, ontogenetic, and other approaches. For example, a relationship between music and language has been proposed, assuming that the evolution of the physical forms of signals is determined by their respective communicative functions [19,20] and that signal production is necessarily associated with a systematic response from listeners. Alternatively, emotional responses to music may be the product of various cognitive processes [21]. Likewise, music can directly access emotional systems without being processed propositionally (from a specific and intentional meaning) [22]. However, this is a nascent field of research, and likely no single approach will fully elucidate the biology and evolution of music.

Regardless of presumptions about the origin and functional character of music, a natural relationship between music and emotion is recognized, either from the assumption that it is a signal shaped by selection to influence the behavior of listeners and transmit adaptive information to their peers [19] or because it has a functional role, e.g., mate selection or other social dynamics, perhaps related to cohesion [23]. Music from various perspectives is considered a stimulus that resonates with basic emotional systems. In humans, many processes of a psychological nature, e.g., deep inner feelings that are difficult to communicate with simple words, are more easily expressed in music [10,24]. It is, therefore, not surprising that “Music is the language of feelings”.

Music is a powerful tool for emotional communication, with trans-specific effects. By definition, music is understood as a product of human creation, corresponding to acoustic, non-linguistic, and intentionally created events structured in time and produced in social contexts [25]. However, this ignores its deep biological foundations and the scope of its attributes. Recent findings and theoretical perspectives suggest that several brain areas that may be critical for the affective emotional processing and appreciation of music are shared homologously and analogously with other mammals [4,26]. Comparative analyses of acoustic signals in non-human animals can shed light on the biological foundations of music, with theories of the emotional origins of music derived from interspecific comparisons and perspectives, such as evolutionary biology [27,28,29]. In addition, our research group identified emotions elicited by music in non-human animals, establishing causal relationships between acoustic features and affective responses [17,18]. This innovative line of research generates new perspectives on music and emotions in non-human animals and the potential to use music as environmental enrichment.

## 2. Emotions in Non-Human Animals

Advances in animal neuroscience, e.g., neuroanatomy, neurophysiology, and cognitive neuroscience, among other related fields, have increasingly demonstrated that animals can experience subjective feelings and mental states previously considered unique to humans [30]. However, there is limited agreement on whether the word “emotion” should be used in the context of non-human species. For many early ethologists and experimental psychologists, emotions were too human and subjective to be included in scientific research involving animals. In recent decades, however, there has been increasing interest in the topic from both ethology and neuroscience, leading to an interest in developing methods that would allow translational studies in both humans and non-human animals [31,32,33,34,35].

The term “emotion” can be defined as a multi-component (subjective, physiological, neural, cognitive) response to a stimulus or event. The conscious, subjective component of an emotion is generally considered its central and key feature; it always has a valence (i.e., positive or negative), may be intense or mild, long lasting or short lived. The nature of the emotion experienced (e.g., sadness, grief, remorse) depends on the nature of the emotive (emotion-producing) event. An emotive event can be external or internal (generated by imagination, memory, or environmental events). An emotive (i.e., emotion-producing) event is usually one that is in some way important to the individual’s goals or relevant to the individual’s well-being. When an emotive event is reliably predicted, that prediction will often produce an emotional response [36]. Although detailed, this definition is considered by some authors to be a cultural construct and not necessarily representative of basic biological structures or processes. LeDoux [37] commented that this type of approach “is simply too human-centered and quintessentially subjective to be applied without ambiguity to non-human animals” [37]. Barrett [38] suggested the need for another definition that is objective to identify, catalog, and study core structures and processes of emotion that can be applied to non-human animals [38].

Several researchers have formulated definitions within this framework, focusing on concrete and objectively identifiable aspects of emotional processing in non-human species [3,39]. Anderson and Adolphs [31] broadly defined emotion as “an internal central nervous system state that gives rise to physiological, behavioural, cognitive (& subjective) responses”. The word “subjective” is kept in parenthesis to indicate that they do not expect that all animals will necessarily produce this component. With this definition, they propose the study of “primitive emotions” in non-human animals, proposing them as similar to, albeit not necessarily homologous with, human emotions. They accept that various phyla and species may have evolved disparate strategies for interacting with conspecifics, the environment, or other animals [31].

From the above approaches, it can be summarized that what humans define as “emotion” is part of the human conceptualization and that other animals have emotional capacities that are sometimes similar to ours and sometimes different based on neural systems of response and anticipation that are deeply specialized, depending on the animal’s niche [40]. This will be important and relevant, not only to our understanding of animals and their welfare but also to our understanding of humans and the behavioral, physiological, and neural underpinnings of our emotions. A comparative approach to emotion should acknowledge and investigate species differences in emotional processing and function.

There is a clear need for emotion investigation, generating more data from multiple studies using various approaches. Some studies, especially those that examined the use of music (a stimulus with an intrinsic emotional value from the human perspective) in the modulation of emotions in various species [17,18,20,41,42], could be valuable.

## 3. Music Is the Language of Emotions

Music can be defined as a human construction of channeled sounds that constitute a spatiotemporally organized sound stimulus producing a complex auditory perception because it is endowed with conscious emotional and figurative states that are aesthetically significant and culturally valued [43]. In that regard, music is a complex elaboration with a structure and meaning based on an emotion perceived by the listener; therefore, it is a language to communicate emotions.

Understanding how music awakens emotional/affective processes is a broad field of research, encompassing evolutionary biology, neuropsychology, neurophysiology, etc., and has led to the emergence of interdisciplinary areas of study, e.g., biomusicology. There is a growing body of knowledge about the emotional processes of the human brain [44], and brain imaging is beginning to reveal the deep brain underpinnings of musical experiences, which we share in a homologous and analogous way with other mammals [45].

For millennia, there has been a close relationship between humans and music, with explanations beyond a purely cultural role, including evolutionary and biological functions. Charles Darwin hypothesized that music may have been a proto-language in ancient times. Others have suggested that musicality is a very ancient capacity that predated and was an essential precursor to human language [46] and that music is based on the prosodic mechanisms of the right hemisphere that allow affective emotional communication through vocal intonation [10]; this is a functional approach that assigns music a communicative and elementary role in the evolution of language. Conversely, others understand music from a cognitive approach [47], asserting that a particular stimulus may evoke different emotions among individuals or even within an individual, depending on context. This implies that the response to music is influenced by individual experience, from learning, and perhaps cultural effects. However, these arguments arise from studies that focus on music, ignore brain neurochemistry that influences emotional experiences, and exclude the possibility of identifying “causal” antecedents of affective states. Perhaps music-induced emotions are fundamentally different from real emotions [48]. Alternatively, music may derive its affective charge directly from brain systems’ dynamic aspects that normally control real emotions and are distinct from, but highly interactive with, cognitive processes [10]. Therefore, the experience of music is highly dynamic and may involve cognitive aspects, such as memory and experience, but can also produce an affective state.

The last integrative perspective is widely supported by evidence from individuals with brain damage [49] or from modern imaging [50]. These studies supported music having multidimensional effects and being processed by widely distributed brain areas [45,51,52]. Music appreciation involves cortical cognitive pathways plus subcortical regions related to emotion induction [45]. Various brain systems mediate emotions, such as anger, fear, happiness, sadness [3], and other social emotions [4], and music can stimulate neuroendocrine functions and systems mediated by norepinephrine and serotonin [53].

Despite scientific evidence indicating how the affective dynamics of music directly modify brain activity, structural characteristics of music that transmit emotion are still under investigation [49,54,55], with more evidence needed to understand causal relationships between music and its effects. For example, electroencephalograms are used to investigate how affective properties of music affect the human brain [56,57], and other neuroimaging and neurophysiological techniques have been used to evaluate subcortical systems relevant to emotion. There is also evidence of excitation of even more primitive subcortical brain regions associated with human affective experience, with increased blood flow in the ventral striatum, amygdala, and other regions associated with emotion and reward [45]. In addition, there is a relationship between emotional arousal during music listening and dopamine release in the bilateral dorsal and ventral striatum [58]. This is a very complex phenomenon that will require an integrative and multidimensional view.

Clearly, a scientific approach to music and emotion is not simple; there are numerous theoretical and conceptual challenges. However, the exquisite sensitivity of humans to sounds and the emotions they evoke seems to be deeply rooted in evolutionary biology. For example, music may enable humans to quickly convey levels of love, devotion, and empathy that would be difficult to achieve through any other form of communication, except perhaps physical contact (although sound is a special form of contact) [10]. People (children to adults) can easily distinguish basic emotions in music [59,60]. Although many mechanisms by which music influences the affective dimensions of our minds have been revealed, this process is so complex that there is debate about the extent to which emotional changes are due to specific musical attributes, e.g., rhythm and melody, or are associated with personal experiences and cultural dimensions [10]. Research on music–emotion relationships is clearly needed.

## 4. Music as a Common Trait between Animals and Humans

The effects of music on human well-being are widely described [61,62]. There may be similar effects in non-human animals based on changes in physiology, cognition, brain chemistry, morphology [4,63,64], and behavioral changes, e.g., decreased anxiety and aggressive behavior [65,66,67]. These findings raise the following questions: What is the biological value of music? And, how does it affect non-human animals?

Music is a complex human elaboration, and until recently, it was considered an artistic product limited to cultural aspects, with no evolutionary or biological components in its manifestation [68]. However, based on modern interdisciplinary research with neural, developmental, and cognitive approaches, it has been demonstrated that music is deeply rooted in biology [68].

Human musicality refers to capacities and tendencies that allow us to generate and enjoy music. Musicality is a stable aspect of our biology; it can be defined as a natural and spontaneously developed trait based on and limited by biology and cognition [68]. However, musicality is not exclusively human, as its basic components can be considered shared with non-human animals [69]. This has led to the emergence of “biomusicology”, with interdisciplinary and comparative research on the effects of music on non-human animals.

From a biomusicology perspective, there are marked similarities between humans and non-human animals in anatomical structures, biochemical signals and their receptors, genes, and behavioral responses that, in humans, have been associated with musicality [69]. Therefore, non-human animals may perceive the basic components of music similarly to humans. For example, the ability to synchronize movement to musical rhythm is a sign of music’s influence on several species [20,70]. However, musicality needs to be better understood from mechanistic, ontogenetic, phylogenetic, functional, and cultural perspectives.

Although all mechanisms involved in music production and perception can be grouped as “the faculty of music” or “the capacity for music”, various components of this capacity may have disparate evolutionary histories. Thus, discussing “music” as an undifferentiated whole or as a unitary cognitive “module” ignores that music has domains (cognitive, emotional, perceptual, motor, etc.), may have many functions (mother–child bonding, mate choice, group cohesion), and may share components with language, speech, etc. [71].

There is a clear need for comparisons of humans and non-human animals to understand interspecific links between biological traits related to musicality, particularly for the physiological and psychological effects of music in humans, as it may have enormous therapeutic potential in other species if it can elicit similar responses. There are effects of music on animals [67,72,73]; however, mechanistic effects on responses, such as stress (that involves complex psychobiological aspects), have not been fully characterized but may be particularly relevant in farm animals.

## 5. Basis for Music Cognitive Processing

Many structural properties of music affect emotional reactions, and music appears to resonate with our basic emotional systems, stimulating affective tendencies encoded within ancient neural circuits often shared with other mammals [10]. The brain is, therefore, the focus of study for the emotional effects of music.

Early work on music neurology highlighted the critical role of the “prosodic” right hemisphere in affective musical appreciation and expression [74,75,76,77,78], whereas many analytical components were associated with the left hemisphere [79,80]. Emotional and affective sensitivity was more strongly associated with the right hemisphere, suggesting a close relationship between affective and musical processes in the brain [77,79]. However, some of the positive affective aspects of musical appreciation activate left frontal areas, whereas negative emotions activate right frontal areas [57], implying complex neural interactions in music processing.

Music is a structurally complex system of notes, chords, beats, themes, and variations, with a complexity that may be similar to language [81]. It has an underlying structure that includes numerous spatiotemporal components. In humans, various brain areas are involved, depending on the aspect, quality, or component of music being analyzed (pitch, temporal organization, timbre, harmony, melody, etc.). Aspects of melodic processing, structure, and musical expectations depend on the function of the superior temporal and frontal cortex [50,81], with regions of the auditory cortex within the right superior temporal gyrus specifically involved in the analysis of pitch and timbre [82]. In response to musical stimuli, blood flow increases in the mesolimbic or reward pathway [81]. The activation of the amygdala, ventral striatum, and hippocampus has also been reported [8,45]; activity in paralimbic and neocortical regions was correlated with the degree of musical dissonance. Human studies using functional magnetic resonance imaging (fMRI) and positron emission tomography (PET) provided evidence that listening to music perceived as pleasant activates specific areas of the brain involved in reward processing, motivation, emotion, and arousal [45] plus areas that regulate autonomic and physiological responses to rewarding and emotional stimuli [81], facilitating the characterization of the neural basis of emotional responses to music.

**Figure 1:**
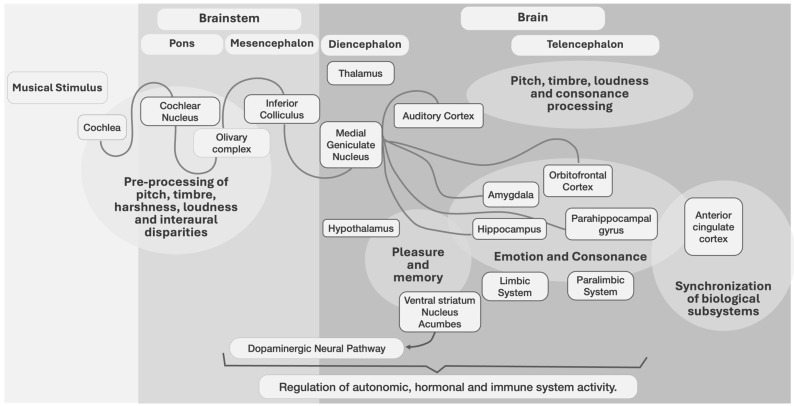
A neurocognitive model of music processing based on [45,81,100,101,102,103,104,105].

Music also affects arousal control systems, e.g., emotional responses controlled by norepinephrine and serotonin [53]. Moreover, dopamine [4,83,84] and cortisol were released in response to all types of music [85]. The cerebellum and basal ganglia, which process rhythm and meter [81], are activated, as well as the hypothalamus (that controls much of the autonomic nervous system, including heart rate and breathing [81]), with systemic effects. Some brain-level interactions for music processing are shown (Figure 1).

Understanding the basic structural aspects of music and its interactions with various aspects of the brain (structure, chemistry, physiological pathways, etc.) provides insights into the potential of music to induce specific neurophysiological responses. Based on limited studies, music may have similar effects in non-human animals. Reward processing areas of the brain are associated with dopamine transmission, and dopamine concentrations increased in chicks exposed to music [10], with a possible effect on reward circuitry.

As physiological changes are a consequence of a stimulus neurocognitive processing, several studies in chickens, rodents, and pigs demonstrated that music, even without optimal acoustic tuning (e.g., stimuli in a standardized human hearing range of 20 Hz to 20 kHz), affects neuroplasticity [86], brain function [87,88], immunity [89,90], and blood pressure [91]. There are also behavioral effects in these and other species, including cats, dogs, pigs, cows (e.g., [41,72,73,92,93,94,95,96]), and wild species (e.g., [42,97,98,99]).

Music is an auditory stimulus whose perception involves multiple brain areas and components simultaneously, determining important emotional and physiological effects. Although the neurophysiology of emotion in response to music is still evolving, with most work conducted on humans, there is impetus to explore how music modulates emotional responses in animals.

## 6. Emotionality as a Component of Animal Welfare Assessment

Welfare is an individual state that simultaneously includes aspects of natural life, feelings or emotions, and biological functioning [14]. However, there is a clear imbalance in the animal welfare literature in the research field and the development of tools to comprehensively assess welfare, with an apparent bias toward measures that assess biological functioning and a lack of research and strategies that address animal psychological aspects. Welfare assessment of non-human animals, especially production animals, has mainly focused on the assessment of resources available to the animal and assessment of indicators of poor welfare, e.g., injuries, body condition, etc., without considering that the absence of these indicators does not necessarily mean that an animal is in a good state of welfare. This approach minimizes the risks of poor welfare, but there are limited tools for assessing positive welfare [106]. Mellor and Beausoleil [106] extended the five domain model for animal welfare assessment to incorporate positive welfare states, resulting in an arguably better and broader approach to evaluating animal welfare.

Animal-based measures of positive experiences are essential, despite the absence of any fully standardized or widely used measure [107]. Observation and semiology can be useful. For example, behaviors such as play are associated with a positive affective state and can be an indicator of positive emotions [108,109]. Qualitative assessment methods have been proposed as an objective way to characterize the global expressivity of animals. For example, the Qualitative Behavior Assessment (QBA) is based on the observation of behavioral signs that reflect an animal’s emotional state rather than unfounded projections of human emotions [110,111]. Rutheford et al. [106] demonstrated that QBA is sensitive to putative experimental alteration of emotional state induced by pharmacological manipulation. Observers blinded to the experimental treatment were able to discriminate between pigs that had received either saline or azaperone (tranquilizer), supporting the biological validity of QBA [110]. The QBA has been correlated with behavioral and other health indicators (e.g., [112,113]), and physiological parameters have supported behavioral expression differences (e.g., [114]) that have promoted its use and growing acceptance in animal welfare science. In addition, the advantage of QBA is that it is a valuable tool for detecting positive emotional states in farm animals [17,18,115,116,117].

Emotional and social communication in non-human animals is an active area of research [118]. Species vocalizations are important for understanding emotion, and various features of emotion in vocalizations generalize across species, yielding specific and testable predictions about the effects of music on animals [41,99,119,120]. Thus, studies of vocalizations in farm animals could lead to the identification of reliable vocal markers of positive states. Despite the fact that there are no fully standardized and widely used measure of good welfare in production systems, animal-based measures of positive affective states should be included, and tools such as vocalizations can be very useful.

There is a large area to be explored related to animal emotions, especially positive emotions, and how they influence animal welfare. In developing assessment strategies, it is desirable to integrate current technological advances, such as artificial intelligence and neural networks, to identify gestures, postures, and other animal-based indicators that can be implemented as an additional tool for the assessment of affective states [121].

## 7. Studies on Music in Non-Human Animals

Several studies have evaluated the effects of music on non-human animals. These have generally evaluated animal behavior and, in many cases, produced conflicting results, even within a species. The key findings are presented below.

In a meta-analysis of 58 studies evaluating music and non-human animals, most studies (62%) used classical music [120]. Although other genres were occasionally used, there was limited diversity in stimuli used, and a criterion for their selection was based on human musical preferences, assuming that classical music has a calming or positive effect. However, chimpanzees had a preference for pop/rock music over classical music [122] in one study and in another study, they preferred Indian or African music to silence [123]. Sparrows had a preference for Bach [124], but only classical music pieces were contrasted.

There is a significant bias among species considered in the evaluation of music, with most animal studies conducted with non-human primates and rodents with auditory characteristics that are either relatively close to humans (orangutans and chimpanzees) or far from humans (mice and rats). This may have contributed to apparent inconsistencies.

Some animals perceive and communicate in distinct ways from humans. Elephants and cetaceans (dolphins and whales) use infrasound (frequencies below human hearing), whereas bats and various rodents use ultrasound (frequencies above human hearing). Although marmosets had no interest in music [42], their natural communication average is approximately three octaves higher than human speech and music, so it is reasonable to assume that exposing animals to music in a frequency range that does not correspond to the species may lead to erroneous conclusions. Filtering out Mozart piece frequencies in the human range (<4 kHz) and presenting them to hypertensive rats was just as effective in lowering blood pressure as the full auditory spectrum [91]; therefore, the only relevant part of the stimulus was >4 kHz [91]. Many loudspeakers may not produce higher frequencies perceived by rats, contributing to failure to detect responses. For some species, music must be beyond the range of human hearing, and even within the range of human hearing, different species perceive different frequencies from those used for human communication and music [125]. In other words, bioacoustics and biological auditory characteristics of the species are relevant to the perception of music and, ultimately, to effects attributed to this type of stimulus.

Most studies did not account for the sensory capabilities of the species tested. However, Snowdon and Teie [41] used this approach with cats, considering the species’ biological characteristics, and observed that when using “cat music”, the animals had a significantly greater interest (orienting, approaching, rubbing the loudspeaker) and significantly shorter latency response compared to their reactions to human music [41]; they concluded that it was important to match music to perceptual capabilities. In Lars’ gibbons, species-typical songs increased animal activity [126]. However, some studies reported positive effects of non-species specific music on various species’ behaviors, including a reduction in “anxious” behavior in gorillas [127]; increased affiliative and reduced agonistic behavior [128] or reduced aggression and exploration with increased social readiness and rest [129] in chimpanzees; reduced stereotypies in elephants [97]; and in dogs, classical music increased sleep and resting behavior but rock music increased activity and barking [72,130,131].

Mixed results have also been reported for farm animals. In cattle, country music improved the voluntary approach to milking stalls, although other types of music were not tested [73,86]. In ponies exposed to a variety of music genres, no significant behavioral changes were observed, but there was a trend toward increased feed intake with country music [132]. In chicks, classical music was beneficial, presenting relatively low cortisol concentrations as a function of dB level, whereas exposure to noise reduced comforting and grooming behavior, impaired learning ability, and increased fear responses [96]. In broilers, classical music ameliorated the negative effects of high stocking densities [133]. In mice, white noise had detrimental effects on gut microbiota, antioxidant activity, and immunity, although music was potentially beneficial [90].

Various studies have been conducted with swine, evaluating music as an environmental enrichment under commercial conditions. One study evaluated piglets’ vocal responses during stressful on-farm procedures and reported no effect of music [134], whereas others reported music increased activity, play, tail wagging, and correlated behaviors [94,95,135]. To our knowledge, only two studies evaluated emotional responses generated by music in pigs (using QBA) and reported that pigs reacted emotionally to different musical adjustments in terms of harmony and spectro-temporal acoustic parameters (beats per minute, spectral deviation, and high-frequency content) [17,18]. In pregnant sows, music induced changes in piglets’ neuroplasticity and improved productivity [86], whereas music reduced agonistic behavior and skin temperature during transport from farrowing to a nursery [136]. The effects of music were studied in sow performance during pregnancy and farrowing/lactation [137] and in mixed or collective housing [138]. Finally, the effects of repeated music stimulation, contrasting short- versus long-term stimuli and noise on stress and immunity, were also reported [139].

In summary, music had a variety of effects that varied among species. There is a conceptual framework based on both the knowledge of species’ natural communication systems and the music structural components identification that can affect emotional states, e.g., calming an agitated animal versus stimulating a relaxed animal. Therefore, the animal-based music concept could lead to consistent and specific effects of music on animals. Knowledge and the appropriate use of music in non-human animals is important in future research if it is to be used in an animal welfare framework.

## 8. Discussion

Why should using music in non-human animals be of scientific interest? Such a question could be asked from neuroscientific and comparative perspectives and approached with a critical view. In humans, music encompasses several aspects, from emotionality to cognition and physiology. Using music in other animal species implies acceptance of similarities, abilities, and complex brain functions that may not be universally accepted. However, increasing evidence of animals’ cognitive abilities facilitates a more comprehensive approach to understanding music from biological and interspecies perspectives. There is scarce research and knowledge about the effects of music on animal emotions, and the application of music in various species has yielded inconsistent outcomes, raising doubts about its usefulness in primates [140], dogs, and chickens [141,142,143,144] based on preferences for silence versus music or the effect of music on behavior. However, such results may be due to methodological or instrumental problems, e.g., type of music, or ignoring auditory capabilities, e.g., auditory range and frequencies that are not preferred [118]. The latter is particularly relevant because it has been reported that different types of music may elicit different emotional responses and that there are important differences among species in perceptual abilities [17]. Therefore, animal species and musical characteristics and animals’ reactions to them must be considered when investigating music in non-human animals.

Emotion is one of the most relevant aspects of the study of music due to its role in neurophysiological effects. Ignoring affective aspects of the musical experience in non-human animals may be inappropriate, as we risk missing some of the most salient and important aspects of animal responses to music. To our knowledge, few studies in non-human animals have focused on it. Some studies have suggested an affective focus in their research [17,18,99], but there is a general lack of scientific context for emotion evaluation in non-humans, despite the scientific recognition of sentience [145] and our close relationship for centuries, with roles in food, clothing, work, companionship, entertainment, and research [146]. Studying emotion in non-human models may improve our understanding of human emotions from biological and evolutionary perspectives [147].

There are many reasons for a lack of knowledge in this field, including the evaluation of emotions in non-human species and methods or instruments used. Emotion has been considered too subjective and too human for scientific research on animals. However, several researchers have focused on concrete and objectively identifiable aspects of emotional processing in non-human species [3,39]. Despite the fact that there is no perfect method for assessing emotional states in animals in relation to music, QBA can effectively be applied [17,18], despite criticisms due to its reliance on human “subjective” perception. This method had high inter- and intra-observer reliability in pigs and other species. Significant correlations with ethogram-based behavioral measures and physiological indicators of stress have been reported (e.g., [148,149,150,151,152]). In addition, other assessments, such as quantitative behavioral measures that are less criticized and more used in the scientific community, did not differ fundamentally in their reliance on human perception and linguistic abilities [111]. Thus, some doubts about the usefulness of QBA appeared speculative; human observers and their perceptual abilities were used as an assessment tool, even for physiological or clinical assessment parameters [111]. Several studies used QBA to assess emotions in horses [153], pigs [110,151], buffalo [154], sheep [155], dogs [156], and elephants [157].

The phenomenon of emotional contagion is an aspect under consideration when a human observer evaluates emotions in animals. The evidence of emotional contagion in this context refers to the fact that an observer contagiously captures an emotional state as a direct result of the perception of the emotional state in another subject or a group of others [32,158]. This mechanism may be a crucial aspect of intraspecific animal communication [158,159] and is considered the most primitive level of empathy in phylogenetic terms, and it seems to be widespread among mammals [32,160]. Consequently, when human observers evaluate the emotions of other animals, they are able to perceive the emotional state through emotional contagion, and the manifestation of the emotions that the human perceives constitutes a consistent evaluation.

Emotional contagion can occur in various forms, e.g., visual and auditory modalities in humans [158], and has been extensively studied in rodents [161,162,163,164]. There is evidence for emotional contagion in birds [165,166], non-human primates [167,168], dogs [169], and pigs [170,171]. Greenall et al. [172] proposed a common emotional system among mammals that may have been conserved during evolution; this suggests interspecific emotional contagion and a bidirectional ability to perceive the emotions of others across species [172]. Furthermore, human cognitive abilities may give humans enormous potential to recognize emotional states in other species. Perhaps domestication had a role in humans’ ability to recognize and empathize with animal emotions [173].

## 9. Conclusions

In humans, music can influence emotional and physiological states, modulating affective and physiological responses that promote well-being. From an interspecies comparative perspective, its effects in non-human animals apparently start from an emotional dimension and neurocognitive processing, modulating various behavioral and physiological responses. Consequently, the potential correct use of music as an environmental enrichment strategy to improve animal welfare requires an understanding of non-human animal emotions from multiple approaches, including various emotional indicators, physiological responses, and brain structural changes (neuroplasticity). Additionally, there is a need to design and adapt music to generate the desired emotional responses that are valuable to them.

## Data Availability

Not applicable.
